# Management of Mandibular Third Molars With an Interradicular Inferior Alveolar Nerve Using Ultrasonic Surgery: A Case Series of Three Patients

**DOI:** 10.1002/ccr3.73510

**Published:** 2026-09-09

**Authors:** Antonio Scarano, Gianluca Nicolai, Ermal Pashaj, Sergio Alexandre Gehrke

**Affiliations:** ^1^ Department of Innovative Technologies in Medicine & Dentistry University of Chieti‐Pescara Chieti Italy; ^2^ Department of Maxillo‐Facial Surgery Università di Tor Vergata Rome Italy; ^3^ Department of Surgery Catholic University Our Lady of Good Council Tirana Albania; ^4^ Department of Biotechnology Universidad Católica de Murcia (UCAM) Murcia Spain

**Keywords:** CBCT, mandibular canal, mandibular nerve, oral surgery, piezosurgery, third molar, tooth extraction, trigeminal nerve injuries

## Abstract

The interradicular course of the inferior alveolar nerve represents a significant challenge for oral surgeon. In this context, ultrasonic surgery may provide a precise and safe alternative for sectioning of impacted third molar roots, particularly in procedures involving mandibular molars with an interradicular course of the inferior alveolar nerve.

## 1 Introduction

The high prevalence of impacted third molars is thought to be related to the evolutionary reduction in mandibular size, which often results in insufficient space for eruption [1]. For these reasons, the incidence of impacted wisdom tooth, which is the last tooth to erupt, has increased, especially in the wisdom teeth [2].

Wisdom tooth extraction is one of the most common procedures in oral Surgery. Frequently, proximity or interaction with the alveolar canal (IAC) has long been a challenge for oral and maxillofacial surgeons.

The main preoccupation for the patient and surgeon is the loss of sensation in the lower lip as a consequence of wisdom tooth extraction. Much research has classified the anatomical relationship between IAN and third molars.

The exact cause of an interradicular course of the inferior alveolar nerve remains unclear.

Theories suggest it could be a developmental abnormality during tooth formation or the result of an ectopic nerve pathway.

Surgical removal of impacted third molars is commonly associated with postoperative inflammatory sequelae [3, 4] including pain, edema, and trismus, as well as transient systemic inflammatory response [4]. These postsurgical sequelae are usually managed by the use of NSAIDs and glucocorticoids [5]. Damage to the inferior alveolar nerve is uncommon but characteristic of this particular surgical procedure [6]. Cone beam tomography (CBCT) examination is essential to know the exact relationship of the wisdom tooth and the nerve. The aim of this study was to describe the management of impacted mandibular third molars with interradicular alveolar nerve managed with the ultrasonic instrument. Piezosurgery allows selective bone cutting while preserving adjacent soft tissues and may reduce the risk of inferior alveolar nerve injury in complex third molar surgery [7].

The aim of this retrospective observational case series is to present three clinical cases involving the extraction of third molars with interradicular nerve positioning and fused roots, performed using ultrasonic surgery.

## 2 Case History/Examination

This retrospective observational study was conducted by reviewing the clinical records of consecutive patients who underwent surgical extraction of impacted mandibular third molars at the Department of Oral Surgery between September 2022 and August 2023. The study was conducted in full accordance with ethical principles, including the World Medical Association Declaration of Helsinki (https://www.wma.net/wp‐content/uploads/2018/07/DoH‐Oct2008.pdf) and the additional requirements of Italian law. The study was exempted from ethical review by the University of Chieti (Italy) as it carries only negligible risk and involves the use of existing data that contains non‐identifiable data. All patients had provided written informed consent for treatment and for the use of their anonymized data for research purposes at the time of care. Written informed consent was obtained from all patients for the publication of this case report and any accompanying images.

## 3 Study Design/Sample

Clinical records of 3 patients (2 men and 1 woman), aged between 22 and 66 years (mean age: 37.7 years), were included. All patients gave their informed consent. The systemically healthy patients were referred to the Oral Surgery Department of the University of Chieti‐Pescara for symptoms referring to the presence of wisdom teeth such as pericoronitis or other space infection. In this study, an ultrasonic device (Surgimoto, Esacrom, Imola, Italy) was used to reduce the risk of the two major complications, namely inferior alveolar nerve injury and periodontal damage to the adjacent second molar, while improving surgical precision. All patients were systemically healthy and were treated between September 2022 and August 2023.

## 4 Differential Diagnosis, Investigations and Treatment

Before wisdom tooth extraction, all patients received periodontal therapy by ultrasonic instrumentation coupled with oral hygiene instructions and motivation. The preoperative panoramic radiograph revealed a mesioangularly impacted left mandibular third molar with a radiolucent band across its roots. The Cone Beam Computed Tomography (CBCT) images indicate radiolucent canal banding across the root fragment as well as expansion of the ligament space, which is consistent with tooth luxation.

The initial appointment included a clinical and radiographic assessment with orthopantomography (OPT), which confirmed the need for unilateral o bilateral extraction of impacted mandibular third molar. Then, before beginning the inclusion processes, all patients signed the informed protocol after being briefed on all aspects of the clinical protocol in order to ensure maximal collaboration. During this phase the teeth were classified in accord the Maglione classification [8].

A total a three clinical record were selected, each presenting with impacted wisdom teeth and an inferior alveolar nerve pathway passing near the root apices. OPT radiographs showed an interruption of the alveolar canal's radiolucent line. Preoperative radiographic assessment was performed using panoramic radiographs for all patients. Cone‐beam computed tomography (CBCT) images, were retrospectively analyzed to evaluate the spatial relationship between the mandibular third molar roots and the inferior alveolar nerve. CBCT imaging allowed precise identification of the relationship between the roots of the mandibular third molars and the inferior alveolar nerve. According to the Maglione classification, three molars were classified as Class 7, characterized by an interradicular course of the nerve and closed roots. In the present study, only these three molars with an interradicular nerve position and closed roots were evaluated.

Prior to extraction, the patients washed their mouths with 0.2% chlorhexidine gluconate mouth rinse solution for 2 min (Curadent Healthcare S.p.A., Saronno, Italy). The facial skin was treated with a 10% povidone‐iodine solution (Betadine, Meda Pharma spa, Italy). Each patient was operated under inhalation sedation with nitrous oxide (Matrix‐Reinhold, Gorgonzola, Italy), and local infiltration anesthesia was administered to the inferior alveolar nerve, lingual, buccal, and retromolar areas by 4% Articaine (Pierrel S.p.A, Milan, Italy) with 1:100,000 epinephrine. All patients underwent a standardized surgical technique done by the same qualified surgeon (AS). After attaining successful anesthesia with an IAN block, a full thickness trapezoidal mucoperiosteal flap with a mesial releasing incision starting from the base of the mesio‐vestibular papilla of the lower second molar, at about 3 mm from the gingival sulcus of the lower first molar, was raised [9]. The flap was extended from the distal sulcus of the second molar to the disto‐vestibular papilla.

**FIGURE 1:**
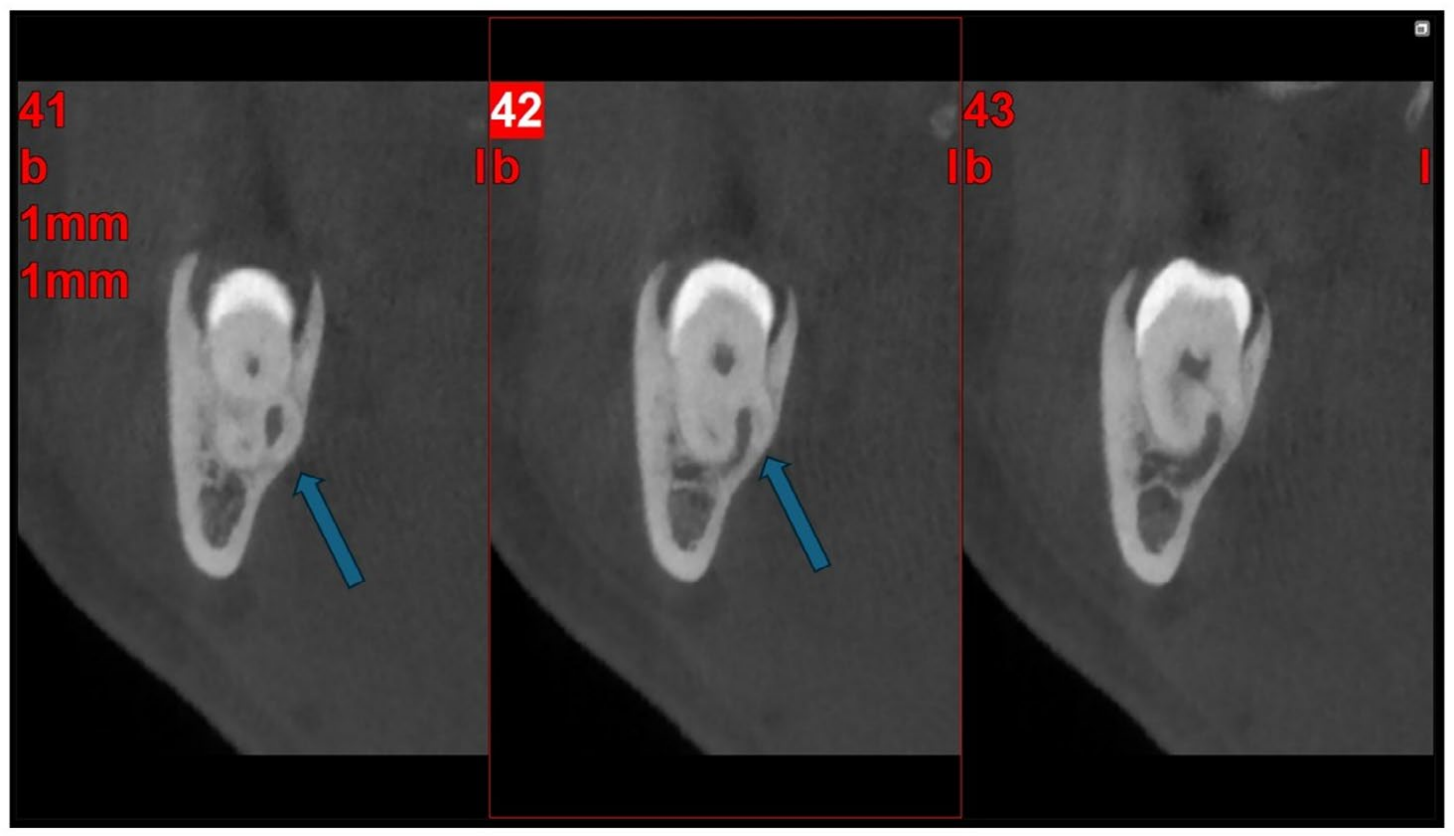
The coronal section of the CBCT shows an interradicular course of the nerve, with the dental roots fused at the apex.

**FIGURE 2:**
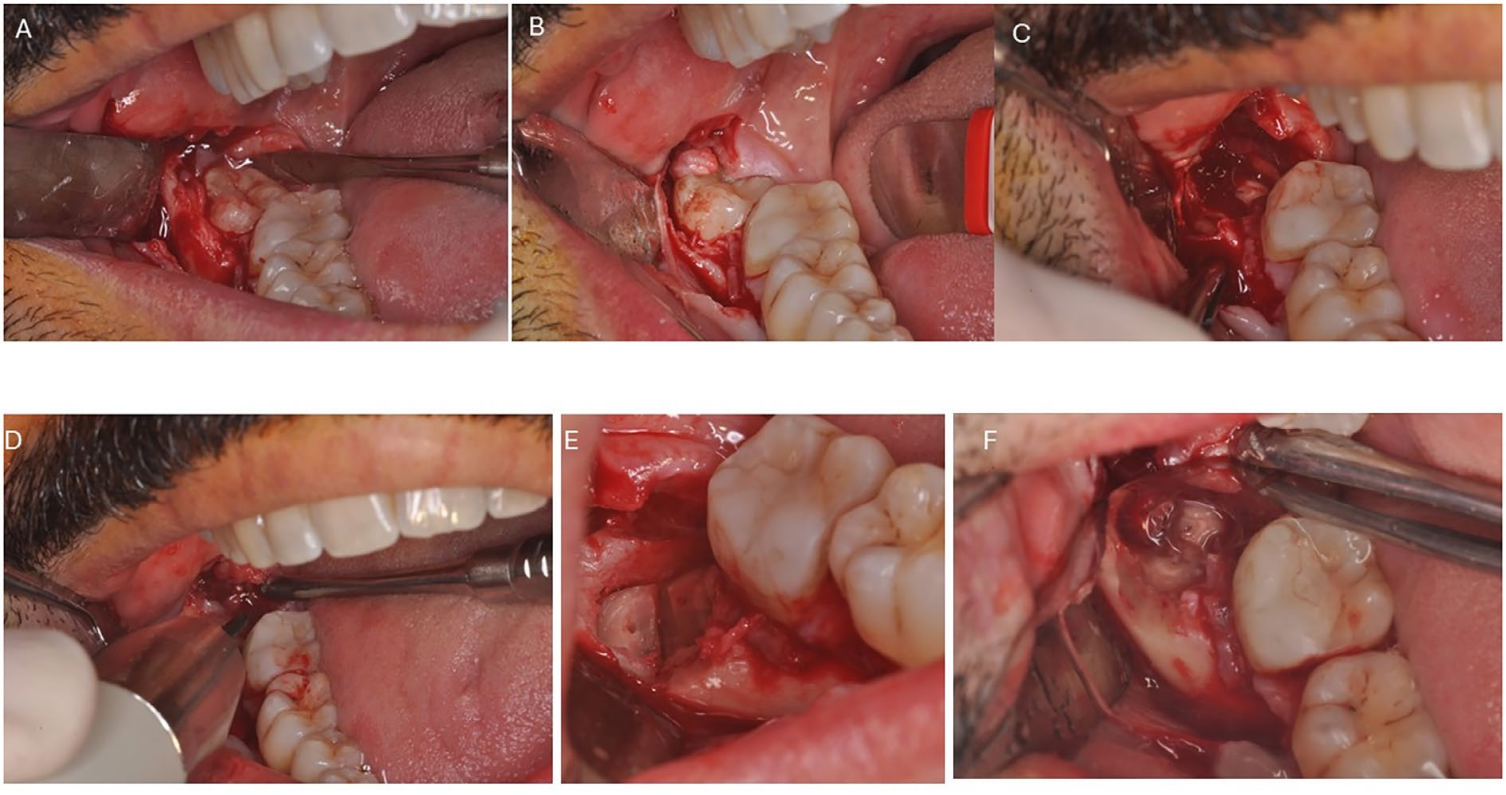
(A) Surgical flap, (B) isolation of the dental crown, (C) removal of the crown, (D) root sectioning using ultrasonic surgery, and (E, F) separation of the roots.

**FIGURE 3:**
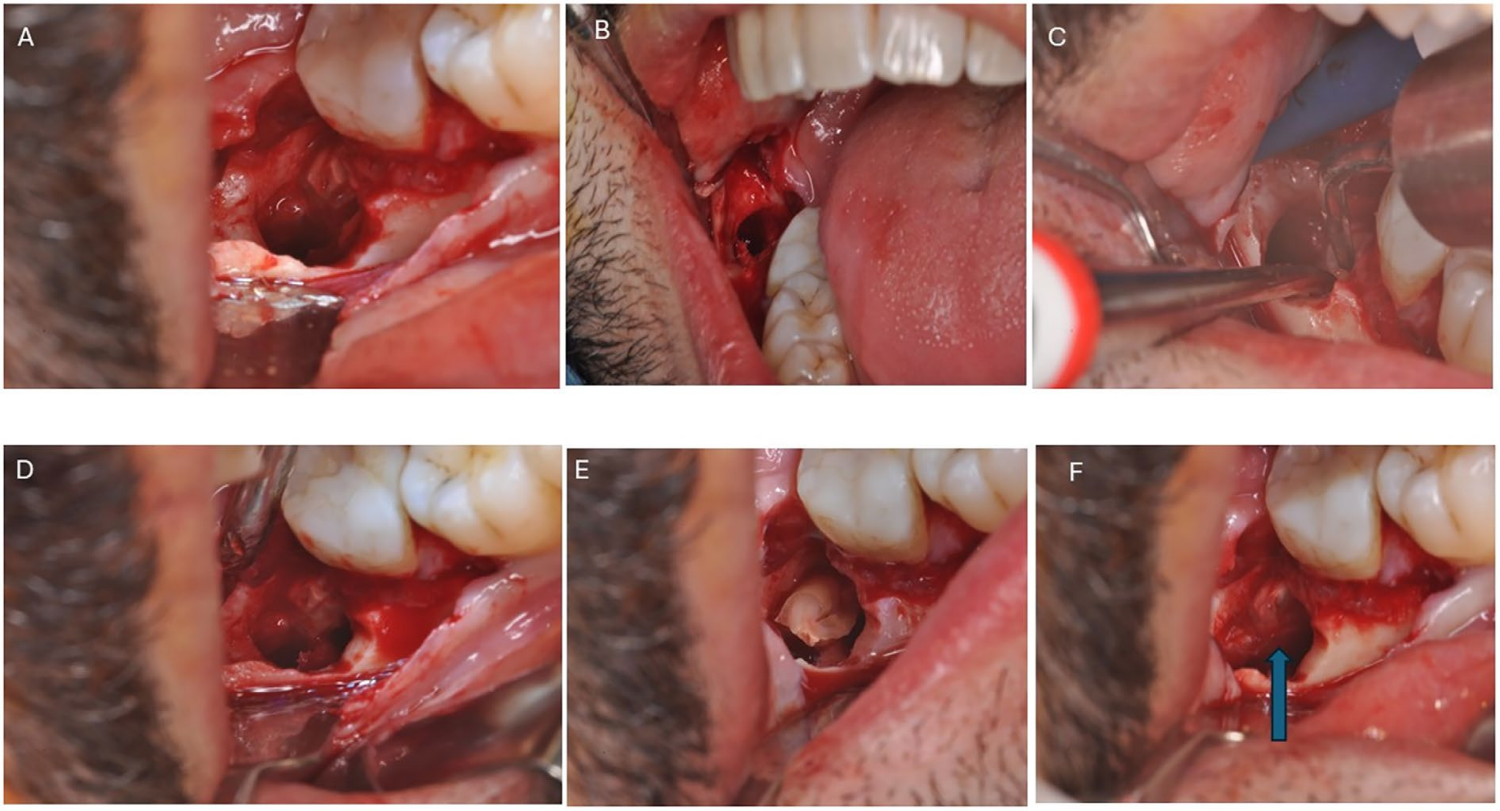
(A) Removal of the distal root, (B) socket after root extraction, (C) luxation of the lingual root, (D–F) various stages of the extraction of the lingual root rotating around the nerve (arrow).

**FIGURE 4:**
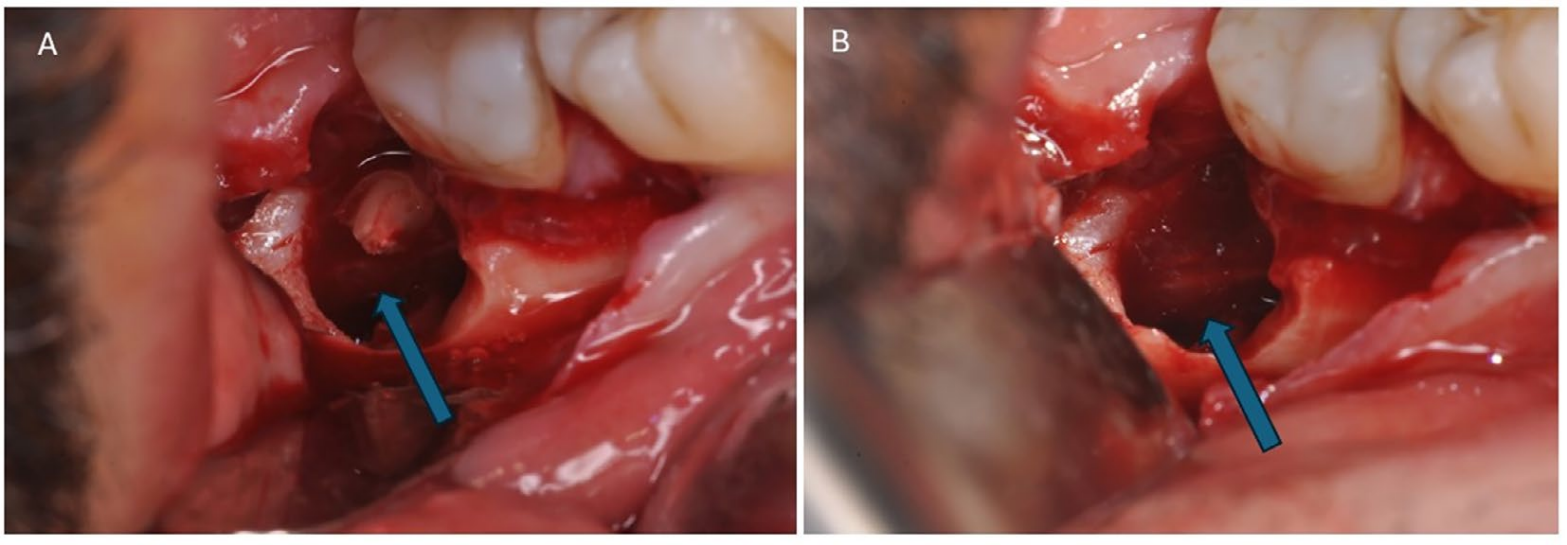
(A) After complete sectioning of the mesiolingual root, it is rotated carefully, paying close attention to the alveolar nerve (arrow). (B) Once the root is removed, the intact alveolar nerve can be observed.

**FIGURE 5:**
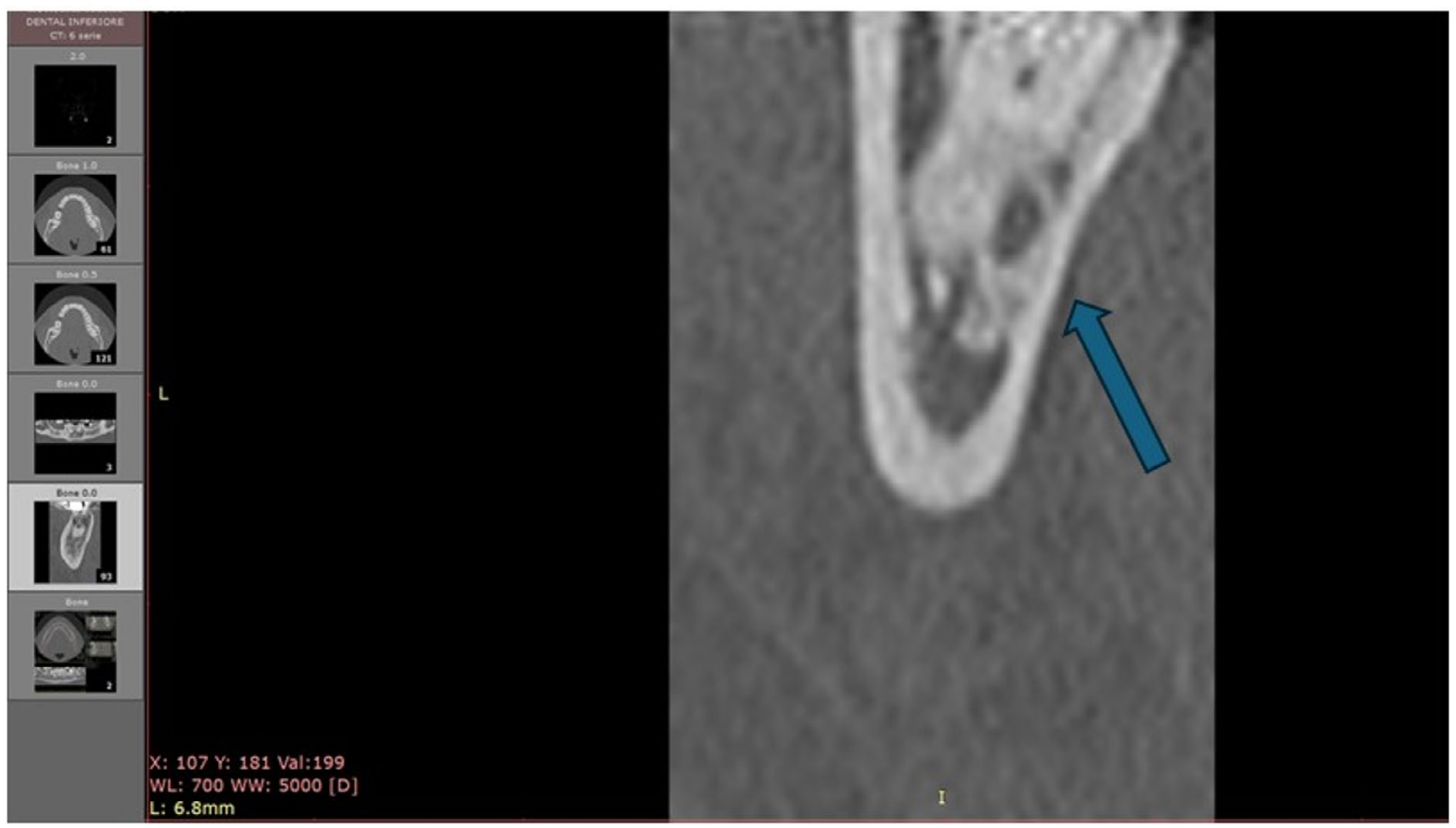
Coronal CBCT section showing the dental apices extending beyond the inferior alveolar nerve, which is located lingually. The roots are fused at the apical level.

**FIGURE 6:**
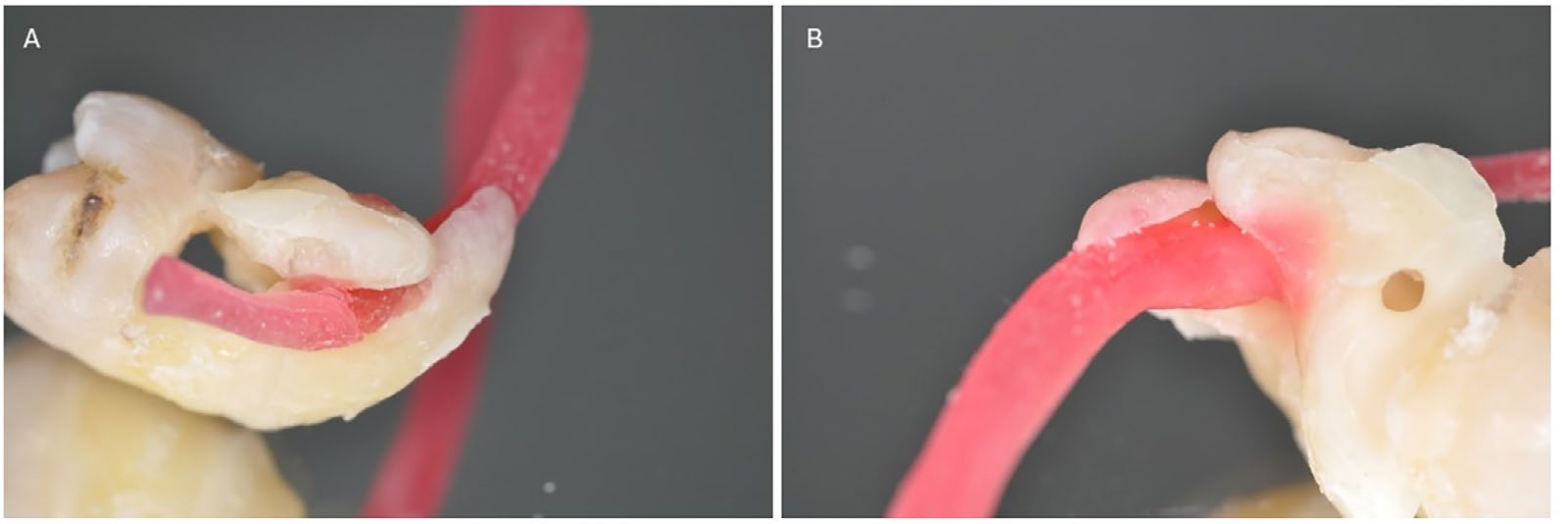
(A) The image shows the wax reconstruction of the ex vivo specimen. The crown and roots, was repositioned using white wax to reconstruct its original anatomy. The course of the alveolar nerve was simulated, showing its path between the mesiolingual and mesiobuccal roots. (B) A distal view of the extracted tooth illustrating this anatomical relationship.

**FIGURE 7:**
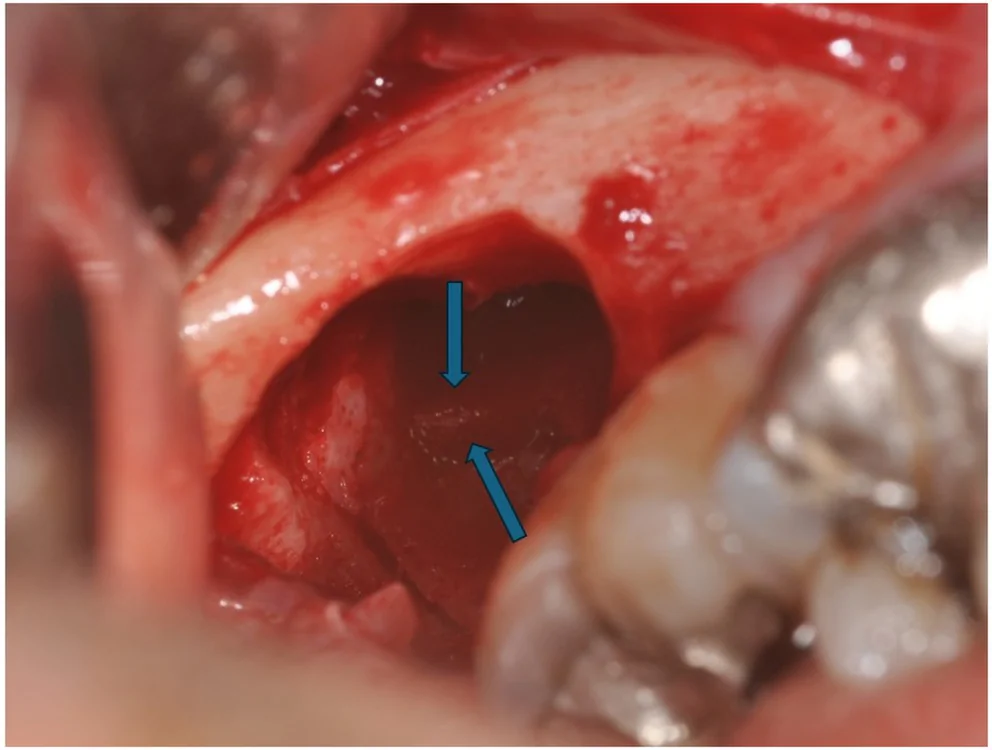
The integrity of the alveolar nerve is evidenced.

The incision was extended to the wisdom tooth's alveolar process, resulting in a disto‐vestibular discharge measuring about 1 cm on the buccal side of the maxillary branch of the jaw. To perform the ostectomy, a tungsten bur mounted on a straight dental handpiece was used under continuous irrigation with sterile saline solution. This exposed the vestibular side of the crown of the third molar until the cementoenamel junction (CEJ) (Figures 1, 2, 3, 4). A straight lever was then used to separate and remove the crown from the root at the same level. After the removal of the dental crown and slight luxation of the tooth, the inferior alveolar nerve appeared clearly delineated and positioned between the roots of the tooth (Figure 2). The levers for extraction permitted dental mobility through the dislocation maneuver, so dislocating the remaining portion of tooth and identifying the inferior alveolar nerve between the roots. The roots were separated by ultrasound and then extracted one at a time to complete the extraction (Figures 2, 3, 4, 5, 6, 7). A Volkmann's spoon curette was used to gently curette only the lateral walls, allowing removal of the residual follicular sac distal to the second molar, while preserving the deeper portion of the alveolus where the inferior alveolar nerve was clearly visible. The flap was repositioned by 4/0 black Poliammide (Assut Europe, Magliano de' Marsi, l'Aquila, Italy) interrupted sutures in order to favor healing by the first intention. The distal part of the flap was left open for wound drainage in the postoperative period. The wound was filled with APG obtained according to the protocol described in previous research in order to reduce pain and take advantage of the antibacterial properties of platelet concentrates [10]. An ice pack was applied for a few seconds to the patient's face for a total time of 20 min.

Neurosensory function evaluation was assessed postoperatively using subjective patient reports combined with clinical examination. Tactile sensitivity of the lower lip and chin was evaluated by light‐touch testing and comparison with the contralateral side. Follow‐up evaluations were performed at 1 week, 1 month, 3 months and thereafter until complete symptom resolution. Recovery was defined as complete resolution of sensory alterations compared with the contralateral side.

## 5 Conclusion and Results (Outcome and Follow‐Up)

In conclusion, ultrasonic surgery proved to be a precise and safe technique for root sectioning, particularly advantageous in complex anatomical scenarios involving the IAN.

Postoperative neurosensory disturbances of the inferior alveolar nerve were retrospectively assessed by reviewing clinical notes recorded during follow‐up visits in the three cases presenting an interradicular course of the nerve. Sensory alterations were classified as temporary when resolution occurred within 3 months after surgery.

The extraction socket showed no symptoms of irritation 3 weeks after the tooth was extracted, and it was covered in normal mucosa. There was no evidence of pus discharge or other inflammatory signs. There was no sign of any complications, such as bone necrosis. Lip and chin sensitivity was assessed by delimiting the area of altered sensitivity with a dermographic pencil, then the percentage was calculated to quantify the area of anesthesia or paresthesia.

Two patients reported anesthesia and paresthesia involving 30% of the chin and lip area of the lower lip and chin for 10 days, which lasted 2 weeks with full sensory recovery after 3 weeks. One patient reported anesthesia followed by paresthesia involving 60% of the chin and lip area after 1 week; these patients achieved complete sensory recovery of the area after 6 weeks. None of the three patients reported permanent sensory impairment, and sensory recovery of the chin and lip was complete (Table 1).

**TABLE 1:** Clinical characteristics, surgical findings and neurosensory outcomes of the three patients.

Patient	Sex	Age (years)	Indication for extraction	Maglione class	Nerve relationship on CBCT	Surgical technique	Postoperative sensory disturbance	Area involved	Recovery time
Case 1	Male	22	Pericoronitis	Class 7	Interradicular IAN with fused roots	Crown removal, ultrasonic root sectioning, separate root extraction	Anesthesia followed by paresthesia	Approximately 60% of lower lip and chin	Complete recovery after 6 weeks
Case 2	Female	25	Recurrent infection	Class 7	Interradicular IAN with fused roots	Crown removal, ultrasonic root sectioning, separate root extraction	Temporary anesthesia/paresthesia	Approximately 30% of lower lip and chin	Complete recovery after 3 weeks
Case 3	Male	66	Recurrent infection	Class 7	Interradicular IAN with fused roots	Crown removal, ultrasonic root sectioning, separate root extraction	Temporary anesthesia/paresthesia	Approximately 30% of lower lip and chin	Complete recovery after 3 weeks

## 6 Discussion

The results of the present study show that transient sensorineural impairment occurred in all patients, the continuity of the inferior alveolar nerve was preserved during surgery, and complete functional recovery was achieved in every case. During mandibular third molar extraction, meticulous surgical technique is essential to avoid injuring the nerve and causing potential complications like permanent numbness or tingling. Before surgery, a thorough evaluation is crucial. Radiographic findings including root darkening and canal interruption may indicate close proximity between the mandibular third molar and the inferior alveolar nerve, increasing the risk of nerve injury during extraction [11]. The contiguity between the third molars and the IAN results in intraoperative neural exposure. This exposure significantly increases the risk of nerve injury, highlighting the importance of careful surgical technique and the potential need for alternative approaches or additional protective measures during extraction [11]. This direct contact increases the risk of nerve injury during surgical extraction or other dental procedures. Mandibular third molar with interradicular alveolar nerve is a rare condition in which the nerve that provides sensitivity to the mandibular teeth and chin (inferior alveolar nerve) coursed through the interradicular space of the mandibular third molar. This anatomical situation can be a challenge for the oral surgeon because it increases the risk of damaging the nerve during surgery. The incidence of IAN injury during LM3 extraction varies widely, with reported rates ranging from 0.2% to 8.4% [12, 13]. Temporary IAN damage occurs in approximately 10.9% of cases, with symptoms lasting from 18 to 180 days [14]. Permanent IAN injury is less common but can have significant long‐term consequences, including persistent neurosensory deficits and psychological impact [15]. The presence of radiographic signs and intraoperative nerve exposure are strong predictors of postoperative IAN injury [16].

Preventive strategies to reduce the risk of IAN injury include thorough preoperative assessment using advanced imaging techniques, careful surgical planning, and the use of alternative procedures such as coronectomy. Coronectomy has been shown to be effective in minimizing the risk of nerve injury by avoiding the removal of tooth roots that are in close proximity to the IAN. Additionally, informing patients about the potential risks and obtaining informed consent based on individualized risk assessments are crucial steps in the management of mandibular third molar extractions. Due to their proximity to the alveolar nerve, which provides sensation to the lower lip, chin, and tongue, mandibular third molars can be challenging to extract safely.

Preventive strategies to reduce the risk of IAN injury include thorough preoperative assessment using advanced imaging techniques, careful surgical planning, and the use of alternative procedures such as coronectomy. Coronectomy has been shown to be effective in minimizing the risk of nerve injury by avoiding the removal of tooth roots that are in close proximity to the IAN [17]. Ultrasonic surgery has been widely adopted in several surgical fields due to its ability to selectively cut mineralized tissues while minimizing damage to adjacent soft tissues [18]. The retrospective design of the study represents an inherent limitation, as data collection relied on the accuracy and completeness of clinical records and no standardized neurosensory testing protocol was applied. The interradicular course of the inferior alveolar nerve represents a rare and particularly challenging anatomical variation encountered during mandibular third molar surgery. In such cases, the use of conventional rotary instruments may increase the risk of nerve trauma. For this reason, ultrasonic surgery was selected owing to its ability to perform highly precise cuts in mineralized tissues while minimizing damage to the surrounding soft tissues [19]. The selective cutting properties of piezoelectric devices contribute to preserving the integrity of the inferior alveolar nerve, even in the event of inadvertent contact between the ultrasonic tip and the nerve. The present study has several limitations that should be considered when interpreting the results. The first limitation is the small number of cases included (*n* = 3), which limits the generalizability of the findings. In addition, the retrospective design of the study may have introduced potential selection and data collection biases. Finally, the absence of a control group precludes a direct comparison with other therapeutic strategies and does not allow a definitive assessment of the effectiveness of the approach adopted.

Nevertheless, these findings may provide a foundation for future prospective studies involving larger patient cohorts and appropriate control groups to confirm the observed results and better define the therapeutic indications of this approach.

In conclusion, the main finding of this case series is the complete reversibility of postoperative sensory disturbances despite the complex interradicular relationship between the mandibular third molar and the inferior alveolar nerve. Although our results support the potential of piezosurgery in preserving neurosensory function, further studies with larger samples are warranted to confirm these preliminary findings and to establish standardized protocols for its use in high‐risk IAN procedures.

## Author Contributions

**Gianluca Nicolai:** investigation, validation, supervision, formal analysis. **Antonio Scarano:** writing – review and editing, resources, investigation, formal analysis. **Sergio Alexandre Gehrke:** funding acquisition, visualization, formal analysis, resources. **Ermal Pashaj:** conceptualization, validation, formal analysis, supervision.

## Funding

The authors have nothing to report.

## Consent

Written informed consent for the publication of the submitted article and accompanying images was obtained from the patients.

## Conflicts of Interest

The authors declare no conflicts of interest.

## Data Availability

The data that support the findings of this study are available from the corresponding author upon reasonable request.
